# Clinical Lycanthropy, Neurobiology, Culture: A Systematic Review

**DOI:** 10.3389/fpsyt.2021.718101

**Published:** 2021-10-11

**Authors:** Sélim Benjamin Guessoum, Laelia Benoit, Sevan Minassian, Jasmina Mallet, Marie Rose Moro

**Affiliations:** ^1^University Hospital Cochin, Greater Paris University Hospitals (AP-HP), Paris, France; ^2^University of Paris, PCPP, Boulogne-Billancourt, France; ^3^University Paris-Saclay, UVSQ, Inserm U1018, CESP, Team DevPsy, Villejuif, France; ^4^Yale School of Medicine (Child Study Center), Yale University, QUALab, New Haven, CT, United States; ^5^University Hospital Louis Mourier, Greater Paris University Hospitals (AP-HP), Paris, France; ^6^Inserm UMR1266, Institute of Psychiatry and Neurosciences of Paris (IPNP), Paris, France

**Keywords:** delusions, psychosis, lycanthropy, zoanthropy, cultural psychiatry, delusional misidentification syndrome (DMS), werewolf, transcultural psychiatry

## Abstract

**Background:** Culture can affect psychiatric disorders. Clinical Lycanthropy is a rare syndrome, described since Antiquity, within which the patient has the delusional belief of turning into a wolf. Little is known on its clinical or therapeutic correlates.

**Methods:** We conducted a systematic review (PRISMA) on PubMed and Google Scholar, until January 2021. Case reports, data on neurobiological hypotheses, and cultural aspects were included. Language was not restricted to English.

**Results:** Forty-three cases of clinical lycanthropy and kynanthropy (delusion of dog transformation) were identified. Associated diagnoses were: schizophrenia, psychotic depression, bipolar disorder, and other psychotic disorders. Antipsychotic medication may be an efficient treatment for this rare transnosographic syndrome. In case of depression or mania, the treatment included antidepressants or mood regulators. The neuroscientific hypotheses include the conception of clinical lycanthropy as a cenesthopathy, as a delusional misidentification of the self-syndrome, as impairments of sensory integration, as impairments of the belief evaluation system, and right hemisphere anomalies. Interestingly, there is a clinical overlap between clinical lycanthropy and other delusional misidentification syndromes. Clinical lycanthropy may be a culture-bound syndrome that happens in the context of Western cultures, myths, and stories on werewolves, and today's exposure to these narratives on cultural media such as the internet and the series. We suggest the necessity of a cultural approach for these patients' clinical assessment, and a narrative and patient-centered care.

**Conclusions:** Psychiatric transtheoretical reflections are needed for complementaristic neurobiological and cultural approaches of complex delusional syndromes such as clinical lycanthropy. Future research should include integrative frameworks.

## Introduction

Culture can affect psychotic symptoms, and psychotic symptoms can be culturally meaningful (1, 2). The Diagnostic and Statistical Manual of Mental Disorders (DSM-5) incorporates structured information to reflect cross-cultural variations in the presentation of psychiatric disorders (3). However, dual neuroscientific and cultural approaches are scarce. In this systematic review, we explore a specific delusional syndrome through both the neuroscientific and cultural perspectives.

Clinical Lycanthropy is a psychiatric syndrome within which the patient has the delusional belief of turning into a wolf. Zoanthropy is a psychiatric syndrome within which the patient has the delusional belief of turning into an animal. Zoanthropy is observed on all continents and animals can be lions, tigers, hyenas, sharks, or crocodiles, for example (4). Wolf transformations have been mentioned since Ancient Times in myths, in popular culture but also in the physician's observations (5). For example, in Greek mythology, Zeus transforms Lycaon, the king of Arcadia, into a wolf. The wolf has negative characteristics of a wicked and evil animal in the Western cultures and more positive ones in the Nordic cultures (6, 7). Wolves still have an important place in today's popular culture, and werewolves still are represented [e.g., *Harry Potter* (8), the series *Teen wolf* (9)].

Clinical lycanthropy is a relevant example of a challenging syndrome at the frontier between neuropsychiatric disorders and environmental, social, and cultural interactions. To which psychiatric disorders is clinical lycanthropy related? What are the neurobiological hypotheses on this syndrome? Is clinical lycanthropy related to a given cultural context? What is the management of this syndrome?

Clinical lycanthropy has been described but not elucidated since Ancient Times. A dual neuroscientific and cultural approach can help to better understand the clinical presentation of patients with psychiatric disorders and help to find relevant avenues of research.

Despite neurosciences still lacking the knowledge to explain the *content* of thoughts, twenty-first-century psychiatry has to take into account these themes and their cultural meaning because they are related to the patients' phenomenology, personal experience, and the patients' and families' storytelling. How would a patient adhere to a specific narrative as a delusional explanation of his/her psychotic experience? Lycanthropy is an example of a syndrome in which culture and environment are involved in the process of the neuropsychiatric disorders' clinical expression.

The objective of this systematic review is: (1) to review the reported cases of clinical lycanthropy; (2) to review the neuroscientific and etiological hypotheses on clinical lycanthropy; (3) to explore the cultural aspects of this syndrome in the medical literature.

## Materials and Methods

A systematic review was conducted according to the PRISMA criteria (10). The research was led on Pubmed/MEDLINE on all available articles until January 2021 with the following words: “werewolf” OR “were-wolf” OR “lycanthropy” OR “clinical lycanthropy.” The inclusion criteria were: case reports of wolf and dog metamorphosis delusions, not restrained to the English, in the psychiatric and neurological literature. The exclusion criteria were: other animal metamorphosis delusions, non-psychiatric and non-neurological conditions referred to as “lycanthropy.” All references were screened individually (abstract ± full manuscript) to identify the case reports. Due to the scarcity of data, the case reports were included, even when they did not include data on the treatment and the outcome. Additional research was conducted on Google Scholar, and by cross-referencing the included studies and prior reviews. The cases from 1920 and earlier were extracted from Blom's review (11). The principal data collected were: age, gender, country, syndrome type, associate psychiatric diagnosis, treatment, and outcome. These data are synthesized in Table 1. There is a risk of bias due to the scarcity of case reports in the literature, and the absence of statistical data, as well as the unbalanced number of publications depending on the country. Additional analysis was led on the neurobiological hypotheses on clinical lycanthropy, and on cultural aspects available in the medical literature.

**Table 1:** Case reports of clinical lycanthropy and kynanthropy.

**References**	**Diagnosis**	**Country**	**Age**	**Gender**	**Treatment**	**Outcome**		**Case n*^**°**^***
(Guessoum et al., 2020) (5)	Schizophreniform disorder	France *Ethnicity: Ivory Coast*	12	M	Antipsychotic *(Low dose Risperidone)*	Remission	Lycanthropy	1
(Marques, 2019) (12)	Schizoaffective disorder (obstructive sleep apnea)	Portugal	41	M	Antipsychotic *(Haloperidol)* Antiepileptic *(Valproate) Psychoeducation and weight lose*	Remission	Lycanthropy	2
	Organic dissociative disorder (obstructive sleep apnea)	Portugal	52	M	Antipsychotic *(Quetiapine, Trazodone) Psychoeducation and hypnotic treatment adjustment*	Remission	Lycanthropy	3
(Groh et al., 2020) (13)	Schizophrenia	Germany	39	M	Antipsychotic *(Risperidone)*	Remission	Lycanthropy	4
(Sayar et al., 2014) (14)	Psychotic disorder not otherwise specified	Turkey	21	M	Antipsychotic *(Aripiprazole)*	Remission	Lycanthropy	5
(Shrestha, 2014) (15)	Chronic psychotic disorder	USA	20	M	Antipsychotic *(Ziprasidone)*	Remission	Lycanthropy	6
(Blom, 2010) (16)	Schizophrenia	Morocco	26	M	Antipsychotic, antidepressants, antiepileptics *(Quetiapine, Valproic Acid)*	Partial remission	Lycanthropy	7
(Younis and Moselhy, 2009) (17)	Psychotic depression *(Severe depressive disorder with psychotic features)*	Iraq or UAE	21	M	–	–	Kynanthropy	8
		Iraq or UAE	26	F	–	–	Kynanthropy	9
		Iraq or UAE	27	F	–	–	Kynanthropy	10
		Iraq or UAE	30	F	–	–	Kynanthropy	11
		Iraq or UAE	72	M	–	–	Kynanthropy	12
	Acute psychotic episode	Iraq or UAE	36	F	–	–	Kynanthropy	13
	Schizoaffective disorder	Iraq or UAE	42	F	–	–	Lycanthropy	14
(Moghaddas and Naseri, 2004) (18)	Psychotic depression	Iran	20	M	Antipsychotic and antidepressant *(perphenazine, fluoxetine)*	Partial remission	Lycanthropy	15
(Nejad and Toofani, 2005) (19)	Bipolar Disorder, mixed type with psychotic feature	Iran	32	M	Antipsychotic *(Risperidone)* Antiepileptic *(Valproate)* ECT	Remission	Kynanthropy	16
(Garlipp et al., 2001) (20)	Schizophrenia	Germany *Ethnicity: Poland*	37	F	Antipsychotic *(Haloperidol)*	Partial remission	Dog-like delusional behavior	17
(Silva et al., 2000) (21)	Psychotic disorder not otherwise specified	USA	46	M	–	–	Partial lycanthropy	18
(Moselhy, 1999) (22)	Undetermined *(several diagnoses received: personality disorder, psychopathic disorder, schizophrenia + DEAF)*	UK	25	M	–	–	Partial lycanthropy	19
	Bipolar disorder, mixed affective episode Epilepsy	UK *Ethnicity: Caucasian*	53	F	Antipsychotics *(Trifluoperazine)* Antiepileptic *(Vigabatrin, Carbamazepine)*	Partial remission	Partial lycanthropy	20
(Rao, 1999) (23)	Psychotic depression, Bipolar disorder	Bangalore, India	21	M	Antipsychotic *(Trifluoperazine)* Mood regulator *(Imipramine stopped and introduction of Lithium Carbonate)*	Remission	Kynanthropy	21
	Psychotic depression	Bangalore, India	17	M	Antidepressant *(Fluoxetine)*	Lack of follow up	Kynanthropy	22
(Verdoux and Bourgeois, 1993) (24)	Psychotic depression Bipolar disorder Obsessive-compulsive personality disorder	France	45	M	1st episode: ECT 2nd episode: mood regulator, antidepressant, antipsychotic *(lithium carbonate, fluoxetine, cyamemazine)*	Remission	Partial lycanthropy	23
(Koehler et al., 1990) (25)	Mania, bipolar disorder	Germany *Ethnicity: Black*	33	M	Antipsychotic	Remission	Kynanthropy	24
(Rojo Moreno et al., 1990) (26)	Psychotic depression	Spain	24	M	Antipsychotic, Antidepressants	Remission	Lycanthropy	25
(Bénézech et al., 1989) (27)	Hysteria and/or mythomania in antisocial personality disorder Alcohol abuse	France	28	M	–	–	Lycanthropy	26
(Dening and West, 1989) (28)	Psychotic depression (doubt on acute organic state)	UK	43	F	Antipsychotic *(Chlorpromazine)*	Remission	Lycanthropy and multiple zoanthropy (cat, dog)	27
(Keck et al., 1988) (29)	Mania, bipolar disorder, cannabis intoxication	USA	18	M	Antipsychotic Lithium carbonate	Remission	Lycanthropy	28
	Schizophrenia	USA	19	M	Antipsychotic	Partial remission	Lycanthropy	29
	Mania, bipolar disorder, obsessive compulsive disorder	USA	17	M	Antipsychotic	Remission	Kynanthropy	30
(Knoll, 1986) (30)	Mania, bipolar disorder	USA	29	M	Antipsychotic	Remission	Kynanthropy	31
	Schizophrenia	–	–	M	–	–	Kynanthropy	32
(Coll et al., 1985) (4)	Psychotic depression	Ireland	66	F	ECT (after inefficient phenotiazines plus antidepressants). Maintenance dose of Antidepressants	Remission	Kynanthropy	33
(Jackson, 1978) (31)	Schizophrenia	USA	56	F	Antipsychotic	Partial remission	Kynanthropy	34
(Rosenstock and Vincent, 1977) (32)	Schizophrenia	USA	49	F	Antipsychotic	Partial remission	Lycanthropy	35
(Surawicz and Banta, 1975) (33)	Schizophrenia (+ hallucinogenic drugs)	USA *Ethnicity: White*	20	M	Antipsychotic *(Trifluoperazine)*	Partial remission	Lycanthropy	36
	Chronic brain syndrome with psychotic symptoms	USA	37	M	Antipsychotic *(Thioridazine hydrochloride)*	Remission (of the psychotic symptoms)	Lycanthropy	37
(Lange, 1970) (34)	Alcohol intoxication	Germany	–	–		–	Kynanthropy	38
(Fodor, 1945) (35)	–	USA	–	F	–	–	Lycanthropy	39
(Schneider, 1920) (36)	Hysteria		–	F	Morphine	Partial remission	Kynanthropy	40
(Deny and Camus, 1905) (37)	Cenaesthesiopathy	France					Kynanthropy/multiple zoanthropy	41
(Rúa Figueroa, 1859) (38)	Psychopathy	Spanish	–	M	–	Death (execution)	Lycanthropy	42
(Morel, 1852) (39)	Lycanthropy	France	–	M	–	Death (starvation)	Lycanthropy	43

## Results

We identified 43 case reports of clinical lycanthropy and kynanthropy (Table 1). Overall, 130 articles were screened in the initial research. Finally, 30 references were included, and 43 cases were identified. In the second part of the results, we present the neurobiological hypotheses of clinical lycanthropy. In the third part of the results, the cultural aspects highlighted in the medical literature are described. In Supplementary Table S1, we documented the other cases of zoanthropy.

### Case Reports: Clinical Presentation, Nosology, Treatment, Outcome

In total, 43 cases of lycanthropy or kynanthropy were identified, including 20 cases of lycanthropy, 4 cases of partial lycanthropy, and 19 cases of kynanthropy. These cases were reported between 1852 and 2020.

**Figure 1:**
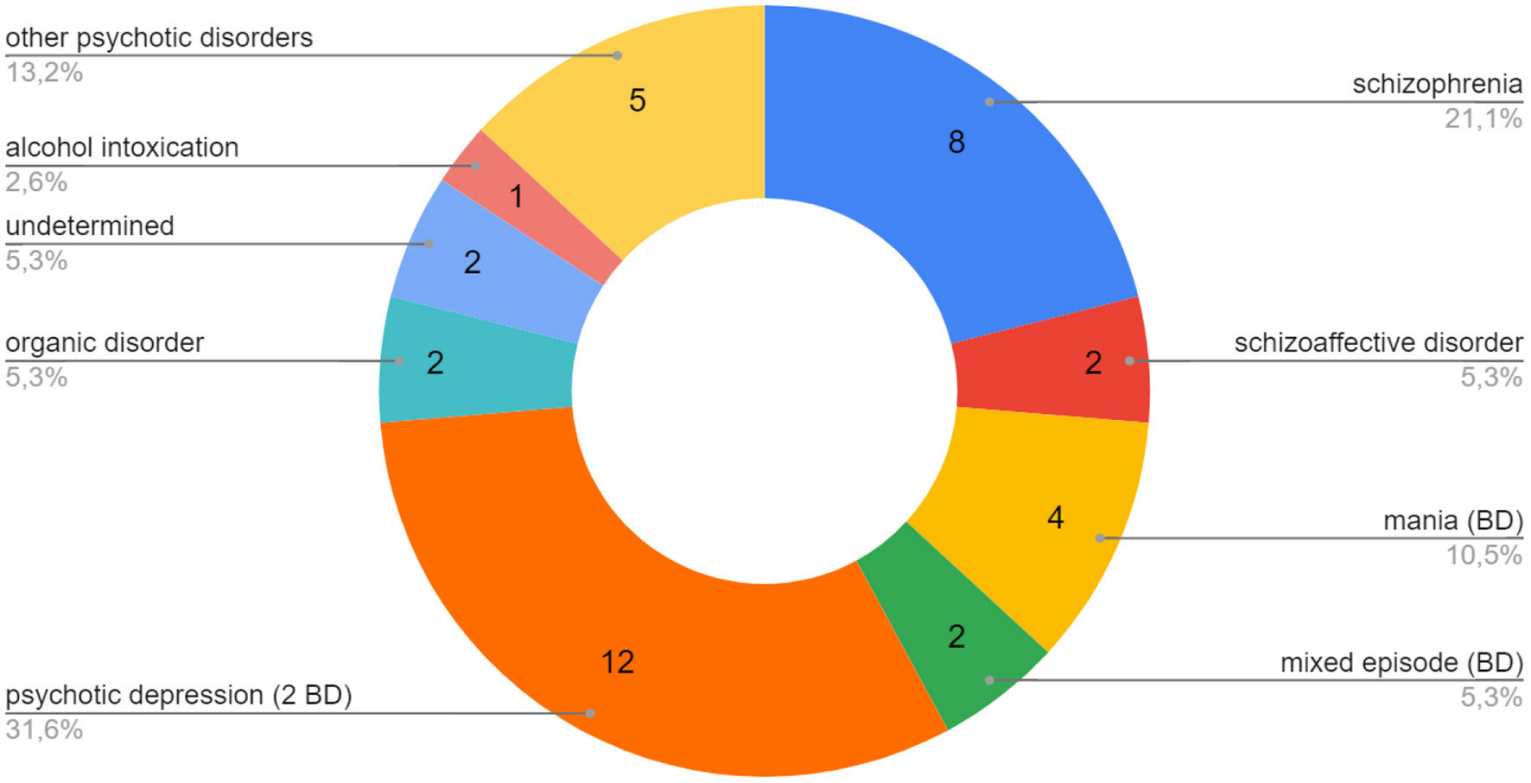
Case reports of clinical lycanthropy and kynanthropy (1970–2020): main diagnoses associated. One case of mania (BD) was associated with OCD, one case of mania (BD) with cannabis, one case of psychotic depression (BD) with OCD, 1 case of mixed episode (BD) with epilepsy. BD, Bipolar Disorder; OCD, Obsessive Compulsive Disorder.

Thirty-eight cases of clinical lycanthropy and kynanthropy were found between 1970 and 2020. The diagnostic repartition of the case reports of clinical lycanthropy and kynanthropy is presented in Figure 1. The majority of the reported cases were psychotic and affective disorders: schizophrenia, psychotic depression, mania (bipolar disorder), and other psychotic disorders. Clinical lycanthropy can occur during a first psychiatric episode of adolescents and young adults (patients n° 1, 5, 6, Table 1). It can also occur in patients with chronic psychosis. Some cases reported were related to neurological conditions, including epilepsy (12, 22, 33). Clinical lycanthropy has also been reported as related to hallucinogenic drugs, alcohol, and epilepsy (40). For example, Keck et al. reported three cases of zoanthropy with electroencephalogram anomalies (29).

Clinically, lycanthropy can be associated with cenesthesic, sensory, visual, and acoustic hallucinations interpreted as related to transformation into wolves. For example, patient n° 18 (Table 1) had hallucinations of hair growth on his face, trunk, and arms, and facial malformations, thus avoiding seeing his face or body. He also believed that his mind was changing into a different mind. Notably, some cases of clinical lycanthropy can be associated with various degrees of suicidality (patients n° 4, 23, 25, Table 1).

The cases reported were treated with antipsychotic medications, plus an antidepressant in case of depression, or a mood regulator in case of bipolar disorder.

### Pathophysiological Hypotheses

#### The Concept of Cenesthopathy

“Cenesthopathy” is a concept that designs pathological bodily perceptions (41). Some case series suggested that cenesthopathies are found in schizophrenia and psychotic depression but may also be partially distinct from schizophrenia (42, 43). Psychopathological studies report that cenesthopathies are related to the patients' “self-existence” feelings, and to strangeness of thoughts (44). Cenestopathy is thought to be more common in adolescent males or middle-aged to elderly women (43). Some cases of clinical lycanthropy may be considered as a cenesthopathy, related to somatosensory aberrations that affect the body scheme (16). According to a study among 30 patients diagnosed with schizophrenia (73% males, mean age 31.5), cenesthopathy is associated with cognitive abnormalities: disturbances of thought initiative and mental intentionality, of receptive speech, and subjective retardation or pressure of thoughts (45). In the case of clinical lycanthropy, the theme of the delusion would be secondary to somatic hallucinations, body scheme illusions and/or perceived changes in physical appearance. Blom reported the case of a 26-year-old man who complained of: increased hair growth, “hardening” of the jaws and facial musculature, changes within the oral cavity and wounds in the corners of the mouth, attributed to the presence of fangs (patient n° 7, Table 1). According to the conception of clinical lycanthropy as a cenesthopathy, the sensory hallucinations would happen first, and the patient would secondarily develop a delusional interpretation of it.

#### The Concept of Delusional Misidentification of the Self

Delusional Misidentification Syndromes (DMS) refer to a fixed, false belief about the identity of a person, place, or object (46). Delusional Misidentification Syndromes include Capgras syndrome, Fregoli syndrome, reduplicative paramnesia, or intermetamorphosis (15). In the Capgras syndrome, the delusion consists of the false idea that someone (often a close relation) is replaced by an imposter (47). Fregoli syndrome is the mistaken belief that some person currently present in the deluded person's environment (typically a stranger) is a familiar person in disguise. The stranger is psychologically identical to the familiar, as if the familiar individual were masquerading as a stranger (48). In the reduplicative paramnesia syndrome, the patient believes that a familiar place, person, object, or body part has been duplicated (49). In the intermetamorphosis syndrome, the patient believes that an individual has been transformed both physically and psychologically (50).

In clinical lycanthropy, the patient believes that he is transformed into a wolf, which can interestingly be conceptualized as a specific type of DMS of the “Self” (15, 51). Some patients believe that they are transforming into wolves while looking at themselves in the mirror (patient n° 1 and 3, Table 1). The 12-year-old pubescent adolescent that we recently described (5) exhibited visual delusions when looking at himself in the mirror, seeing his eyes changing colors and his teeth lengthening. His symptoms were different from another rare type of DMS, the mirror delusional misidentification syndrome, also called the mirror sign, in which the patient treats the mirror image as a separate individual (46). Mirror delusional misidentification and Capgras syndrome could both be related to facial recognition impairments (46).

There is a relationship between DMS and right hemisphere dysfunction (52, 53). A review on lesion-related DMS reported localization to the right hemisphere and frontal lobe, although wider networks may be involved (54). Right hemisphere anomalies would play an important role in neuropathologies of the self (55). To our knowledge, there are no neuroscientific studies on zoanthropic syndromes.

#### Clinical Overlap With Other Delusional Misidentification Syndromes

Our review shows that there is an important overlap between clinical lycanthropy and other DMS (for example patients n° 15, 16, 19, 27, 34, Table 1). A 20-year-old patient with psychotic depression experienced delusions in which other people transformed into wolves and leopards (n° 15, Table 1). A 32-year-old man with bipolar disorder experienced kynanthropy, thought his wife and daughters had transformed into dogs and experienced Cotard syndrome [i.e., delusion of “negation,” within which the patient can deny being alive, or having guts (56)] (n° 16, Table 1). A 43-year-old woman with psychotic depression thought that she transformed into a wolf and other animals (cat, dog), and she said her husband appeared as the devil (n° 27, Table 1). A 56-year-old woman with schizophrenia experienced clinical kynanthropy, Capgras delusions, and erotomanic delusions (n°34, Table 1). The frontier between self-delusion and intermetamorphosis is porous, given that some patients with lycanthropy also have delusions of other person's transformations.

The clinical overlap between various DMS may suggest the hypothesis of shared mechanisms between clinical lycanthropy and other DMS. Patient n° 34 (Table 1) rapidly remitted the kynanthropic delusion thanks to antipsychotic medications but she then suffered from delusional misidentification of strangers and friends. Two days after a second episode of kynanthropy, she experienced again a Capgras syndrome, she thought her psychiatrist was an impostor. Arisoy et al. reported the case of a multiple DMS (reduplicative paramnesia, intermetamorphosis, reverse-intermetamorphosis, misidentification of reflection, and Capgras syndrome) in a 17-year-old adolescent (57). Overall, these clinical overlaps of DMS may suggest a pathophysiological overlap and may support the inclusion of clinical lycanthropy within the DMS syndromes.

#### Visual Processing Anomalies and Belief-Evaluation System

A “two-factor theory” has been suggested as a possible explanatory hypothesis for lycanthropy (12, 15). Some patients with clinical lycanthropy experience visual delusions (for example, patients n°1, 2, 3, 36, Table 1), suggesting an anomaly in visual sensory processing or integration. Theoretically, the inappropriate perceptions would need a second error of the patient for the delusion to be effective: the inappropriate perception would then be treated erroneously. The belief-evaluation system would ineffectively treat the inappropriate perception, preventing the false thought to be rejected.

These delusions would be the result of two factors: a mismatch processing sensory information of the self and impairment in the belief-evaluation system.

#### Sleep and Clinical Lycanthropy

##### Chronobiology and Circadian Rhythms

Clinical lycanthropy is sometimes associated with the patients claiming to transform into wolves depending on the lunar cycles (for example patients n°1, 36, Table 1).

Erren and Lewis hypothesize that some rare werewolf cases may be due to manic and aggressive behaviors that would have been triggered by moon-associated signals (58). The 4 hypotheses of Erren and Lewis concerning lunar effects are the following ones: “(i) some as of yet non-measurable signal or undefined geoelectric/geomagnetic physiological receptor, (ii) Moonlight affecting sleep and/or circadian rhythm, (iii) imprinting susceptibility at birth, (iv) lunar periodic growth of, and subsequent consumption of or exposure to, psychotropic agents” (58).

Wehr showed that an association of some rapid-cycling bipolar disorders with lunar cycles is plausible, studying the lunar cycles and the circadian rhythms of 17 patients with rapid cycling bipolar disorders (59). Hypothetically, it may also be related to the lunar-periodic association of the body temperature rhythms. A retrospective study on 13,067 patients in China reported that psychiatric admissions for schizophrenia show lunar periodicities, with peaks in the first quarter, followed by the full moon and lowest at the new moon. The patients with paranoid schizophrenia were more likely to be admitted at the full moon, using unspecified schizophrenia as a reference (60). On the contrary, a study on 17,966 patients with any psychiatric disorders in Switzerland found no connection between lunar cycles and psychiatric admissions (61). The lunar cycle may have a relation with sleep-wake patterns (62). Evidence of moon-associated sleep symptoms is low, as well as a hypothetical lunar cycle-associated delusional syndrome.

##### Obstructive Sleep Apnea

Patients n° 2 and 3 (Table 1) are two cases of clinical lycanthropy (one with schizoaffective disorder and mild mental retardation, the other with organic dissociative disorder) who also suffered from Obstructive Sleep Apnea (OSA). Marques (12) hypothesizes that awakenings or hypoxemic periods induced by obstructive sleep apnea may contribute to lycanthropic delusions. The two patients' delusions disappeared after treatment adaptations, psychoeducation, plus weight loss for the one, and hypnotic treatment adjustment for the second. In the literature, there are other cases of psychotic spectrum disorders comorbid of OSA, such as psychotic depression (63), schizophrenia (64), or other psychotic symptoms (65, 66), which improved or remitted after continuous airway pressure treatment.

### Cultural Aspects

#### Transcultural Aspects

**Figure 2:**
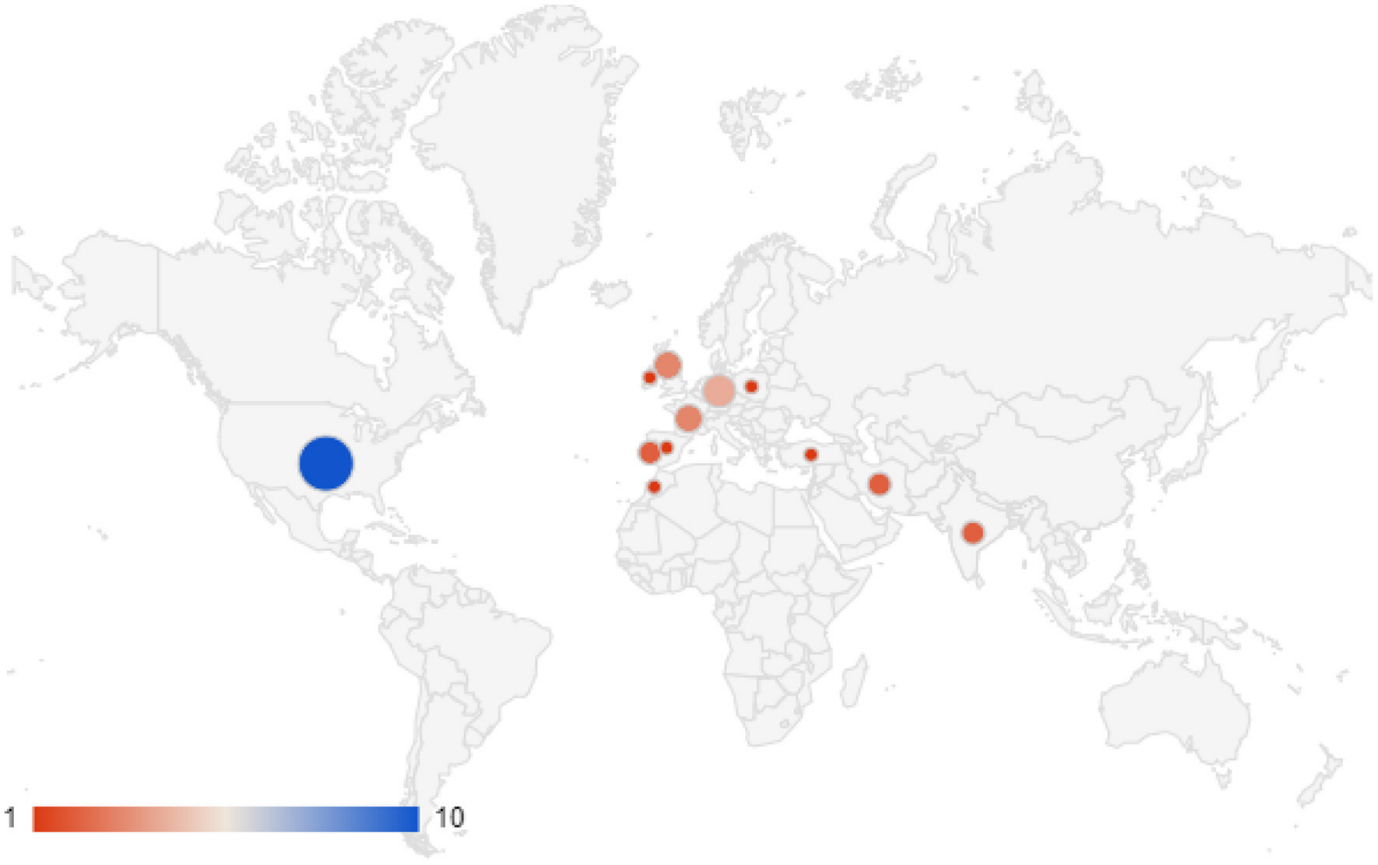
Case reports of clinical lycanthropy and kynanthropy in the scientific literature (1970–2020): geographical repartition.

The cases of clinical lycanthropy and kynanthropy were reported in the following countries (Figure 2):

- the USA,
- Western Europe (Portugal, Spain, Ireland, UK, Germany, The Netherlands), and the patients originated from these countries, or Africa,
- Turkey,
- Iran,
- India.

Some authors suggest that clinical lycanthropy is a culture-bound syndrome (67). From an anthropological perspective, animal transformation does not have the same meaning depending on the culture. In Western cultures, animals and humans are separated ontologies, whereas the borders between animals' and humans' interiority and physicality exist differently in other cultures (68). Also, concerning psychotic symptoms, cultures have an impact on the experience of hallucinations, and hallucinations can be culturally meaningful (1).

The symbolism of the wolf is different among cultures. In Western Europe, the wolf is associated with negative aspects, perceived as dangerous or even evil (6). On another hand, the Nordic Europe traditions associate the wolf with positive characteristics (7). Scandinavian warriors, the *ulfhednir*, ate wolves and dressed in wolf fur before fights (6). In the Middle East, Younis and Moselhy explain that the wolf has a dual paradox meaning of impurity and loyalty. In the eight cases of lycanthropy and kynanthropy they reported, most of their patients endured psychotic depression, with delusional ideas of guilt and dirtiness, thus identifying themselves with the negative meaning of the dog (17). In India, a case of kynanthropy was culturally attributed to black magic in Bangalore province (patient n° 22, Table 1), and the traditional treatment was to vomit to remove the effects of the “evil eye.” We also spotted several cases in which evil was evoked within the patients' discourses, in Western Europe (patients n° 4, 17, 27, Table 1). In Zimbabwe, we found a report of voluntary transformation in a jackal in a “lycanthropic ceremonial” in 1933 (35). In Arabic medicine, Avicenne describes the mania lupina and mania canina (Avicenna, Canon, lib.3, fen1, tr.4, ch.15).

Keck et al. reported that lycanthropy was also reported in nineteenth-century Japan (69). In Japanese culture, the fox has a more important place and cases of belief in fox transformation or possession have been reported (70). The belief of possession by foxes (*kitsunetsuki*), and the belief of possession by dog-like mythological beings (*inugami*) had been existing in the Japanese culture, but when the Japanese adopted Western medicine, they believed less in animal possession and rather considered it as a mental illness (71, 72). The delusion of possession by a fox was described as the most common animal possession in some populations of patients in Japan (73). These examples show that the content of psychiatric symptoms, such as the type of animal-related delusion, is influenced by the cultural background.

#### Culture and the Narration of the Delusion

The cultural factors implicated in the delusional syndromes are still insufficiently understood. Yet cultural factors do influence the content of delusion, as illustrated by these case reports, and consequently, these delusions are part of the patients' narrative, which clinical psychiatry deals with. Lack of cultural understanding may prevent clinicians from adequately responding to patient distress (74). The relationship between delusional types or themes and the pathophysiological mechanisms are still unelucidated, despite some studies pointing out that specific neuropathological mechanisms may correspond to precise delusional expressions (75). Environmental stress factors of clinical lycanthropy can be a dog bite, leading to delusional interpretation (23), which suggests a relationship between the nature of traumatic events, and the clinical expression and content of the neuropsychiatric disorder.

#### Lycanthropy, Internet Culture, and Internet-Mediated Delusions

Digital culture allowed patient n°1 (Table 1) to immerse into this mythical imagination *via* series and online searches. Day and night, he watched series and websites related to wolves and werewolves. Two other patients experienced clinical lycanthropy delusions as related to series, or online research, that they used to find an explanation for their hallucinations (patients n°6 and 7, Table 1). Another patient feared watching movies that could interfere with his lycanthropic delusions (patient n°18, Table 1). Earlier, in 1975, Surawicz and Banta reported the case of a 20-year-old patient with clinical lycanthropy after he saw the movie “The Exorcist” (patient n°36, Table 1). Research on the link between delusions and the internet remains sporadic (76). In today's adolescent culture, werewolves are still represented in literature, such as the character Remus Lupin in JK Rowlings' *Harry Potter* (8), in *Twilight* (77), or in the series, such as *Teen Wolf* (9). Digital technology, the internet, social networks, series, video games, and virtual reality games are now a main part of the culture. These cases suggest that studying the consequences of digital technologies on adolescents' mental health and assessing their use in daily clinical practice is relevant.

## Discussion

This review identified:

1. Forty-three cases of clinical lycanthropy and lycanthropy, mainly associated with psychotic spectrum disorders (schizophrenia, bipolar disorder, psychotic depression, and other psychotic disorders). These delusional syndromes clinically overlapped with other delusional misidentification syndromes.

2. Several neurobiological hypotheses on clinical lycanthropy that may interact: cenesthopathy, delusional misidentification of the self, circadian rhythms dysregulation, abnormal sensory perception and integration, impairments in the belief-evaluation system, right hemisphere anomalies.

3. Cultural aspects: if zoanthropic syndromes are reported in various continents, clinical lycanthropy has mainly been reported in Western countries, and may be a culture-bound syndrome. Wolf transformation delusion may be the result of a neurobiological psychiatric disorder happening in the specific context of Western cultures, myths and stories on wolves and werewolves, and today's exposure to these narratives on cultural media such as the internet, and the series.

The strengths of evidence are low for these results.

### Lycanthropy in History

The oldest known writings mentioning lycanthropy are found in the Greek myth of Lycaon, the King of Arcadia (78). The most famous case of zoanthropy is King Nebuchadnezzar II (605-562 BC) who endured delusional beef transformation delusions. The animal transformation of Nebuchadnezzar is mentioned in the Bible (Daniel 4; 29–31). In ancient Egypt, Anubis, the god of death and the inventor of embalming, is represented with the head of a jackal or, more precisely, an African golden wolf (79). Interestingly, in ancient Egypt, Anubis and the lunar circle are represented together, showing a very ancient association of men-wolves with the moon (80). In Byzantine times, physicians explained lycanthropy as a type of melancholic depression or mania (81). In medieval and Renaissance times, lycanthropy was often attributed to satanic forces. Kynanthropy was described in Byzantine late Antiquity, for example in the city of Amida (eastern Turkey) (82). Aetius of Amida devotes an entire chapter to lycanthropy and kynanthropy, which underlines a clinical significance in these times (7). In medieval and renaissance times, lycanthropy was often attributed to satanic forces (11). Beliefs in werewolves and other zoanthropic phenomena are still very present in some cultures, for example in Haiti, where the werewolf refers to women transforming at night and sucking the blood of young children (83). Transformation into an animal can happen in the cultural context of possession, in rituals and traditional therapies (84, 85). The animal is then both an incarnation and a totem.

### Other Zoanthropic Syndromes

Lycanthropy and kynanthropy are specific forms of zoanthropy. Zoanthropy is observed on several continents, and animals are as variable as lions, tigers, hyenas, sharks, or crocodiles (4). During the review on lycanthropy, we also found other cases of zoanthropy: snake transformation in Lebanon (67), pig in India (86), wild boar in Germany (87), cow in the Middle East (17), frog and bee in Germany (88), gerbil, bird, cat, rabbit, tiger in the USA (29). The environmental and cultural context is a factor that influences the content of the delusion. For example, Garlipp et al. reported the case of a 43-year-old man with a history of bipolar disorder, who believed to be transformed into a wild boar, during an alcohol intoxication (87). This delusion happened after real contact with those animals.

Keck et al. reported two cases of ailuranthropy, or cat transformation delusion, one in a 37-year-old woman with a bipolar disorder that remitted after treatment, the other in a 24-year-old man with atypical and resistant psychosis, who believed since 13 years, without interruption, that he was a cat, lived with cats and had sexual activity with cats. Reports of ailuranthropy are very rare compared to lycanthropy, despite the important place of this animal in human cultures.

### Psychodynamic Hypotheses

Some cases of lycanthropy and kynanthropy we reported were associated with feelings of guilt, dirtiness, or evilness, and the idea of being transformed into a symbolically negative animal may be related to these depressive and delusional symptoms. Also, during adolescence, the bodily and identity transformation during this period of life may interfere with the delusional experience of lycanthropic transformation (n° 1, 22, 28, 29, 30, Table 1).

### Non-psychiatric Cases of Lycanthropy

“Werewolf syndromes” are also found in medical publications referring to non-psychiatric diseases: rabies, lepromatous leprosy, congenital hypertrichosis, gingival fibromatosis with hypertrichosis syndrome, congenital erythropoietic porphyria (40, 89–93). In these cases, the word “werewolf” is used due to the appearance of the patient. For example, gingival fibromatosis with hypertrichosis syndrome is a heterogeneous group of diseases in which the patients suffer from excessive universal hair growth and gingival overgrowth, which is seen as a wolf-like body transformation. Congenital erythropoietic porphyria (Gunther's disease) is characterized by skin photosensitivity and fragility leading to severe scarring of the face and hands, hirsutism, red discoloration of teeth, finger-nails, and urine, and stunted growth (92). These syndromes are somatic conditions, but they have a psychological and social impact (90). The treatment is the medical treatment of the disease itself.

### Cultural and Cognitive Correlates of Delusion

The existence of cultural correlates of delusion experience and expression and their influence on clinical assessment in biological psychiatry are admitted but poorly known (1, 2). This aspect is rather studied in other approaches, especially transcultural psychiatry. The cultural context influences the delusional content of schizophrenia (94, 95). A study compared the content of patients' delusions and hallucinations in three groups (Pakistanis of 1st and 2nd generation living in Britain, Pakistanis living in Pakistan, and British White living in Britain) and surprisingly observed more differences between Pakistanis living in Britain vs. in Pakistan than between Pakistanis vs. White living in Britain (96). Also, the statement of the DSM that the diagnosis of a delusion should exclude shared cultural beliefs is questionable (97). Clinical lycanthropy is an example of a psychiatric syndrome for which the psychiatrist needs both a neurobiological and cultural perspective to understand the disorder. Building a model that would integrate cognitive aspects and cultural factors is a challenge. Various cognitive models aim at explaining delusions (98), delusions are considered to be “pathological beliefs” explaining abnormal perceptions. Some factors would explain the content of delusions while others would explain their presence (99). Some cognitive models retain two main factors: perceptual abnormalities, and mistakes in the beliefs related to these perceptual experiences, through cognitive biases (98). Cognitive biases play a role in the development and the maintenance of delusions. Some factors may influence the cognitive process of delusions (for example, trauma-related memory, or state of negative affect, can influence information processing) (98). Persecutory delusions are much studied (100), while scientific knowledge about DMS remains scarce. The content of delusions is often personally, environmentally, and culturally relevant (96, 101). For example, the type of delusion may be influenced by the conceptions of the self and the type of family relationship across countries (102, 103). Our review supports that cultural factors, including collective beliefs and fictions, may influence the content of delusions, possibly by contributing to the cognitive biases described in the cognitive models of delusions.

### Outlines for an Integrative Model

**Figure 3:**
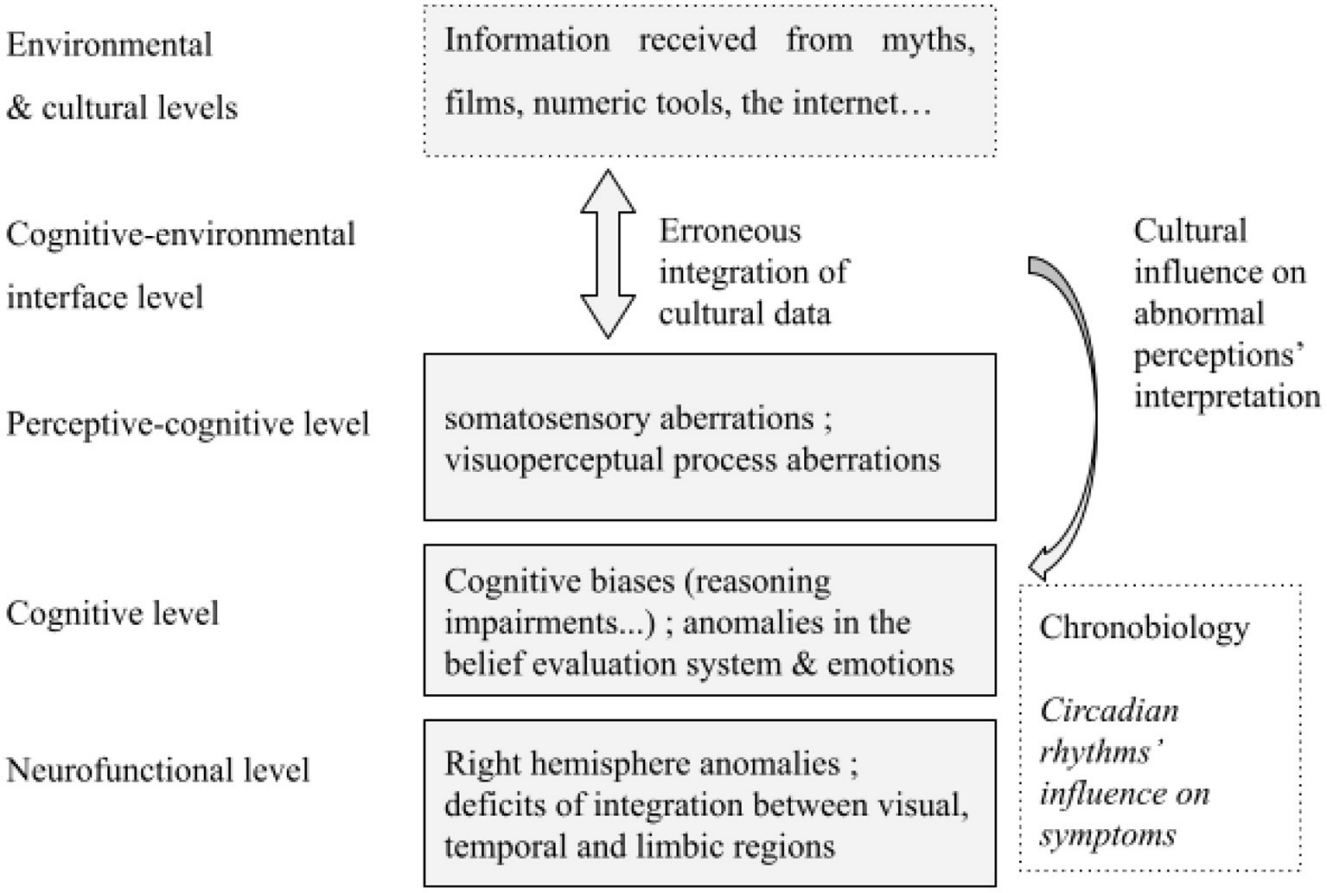
Multi-level hypotheses for clinical lycanthropy syndrome.

Several levels of understanding of clinical lycanthropy can be proposed. The difficulty lies in the theoretical splitting of different yet complementaristic hypotheses and aspects, that include neurobiological, and anthropological hypotheses (Figure 3).

### The Psychiatric Management of Clinical Lycanthropy

At the clinical level, the psychiatric management of clinical lycanthropy should include research of other delusional misidentification syndromes, given the important clinical overlap we reported. Also, some neuropsychological tests are pertinent besides the acute episodes.

The delusional theme of animal transformation may be both embedded within the patient's psychiatric disorder, associated with mood and psychotic symptoms, and related to the cultural meaning of lycanthropy and the environmental exposure of the patient to these narratives. The use of the internet and numeric tools should be assessed. Furthermore, the cultural and linguistic features of the patient should be assessed, to adapt the access to care and psychotherapy to the patients' cultural background (104–107).

Given the occurrence of lycanthropy in the context of neurological conditions and intoxications, careful consideration should be given to neurological exams, including brain imaging, EEG, and toxicological exams (108). Screening sleep disorders is also pertinent.

Antipsychotic medications may be an efficient psychopharmacological treatment for this rare transnosographic delusional syndrome. Mood disorders are highly associated with clinical lycanthropy. In these cases, patients remitted of clinical lycanthropy thanks to antidepressants plus antipsychotics in case of psychotic depression, and mood regulators plus antipsychotics in case of bipolar disorders (Table 1).

Psychotherapy is indicated in mood and psychotic disorders, which are associated with clinical lycanthropy. To our knowledge, there is no scientific evidence regarding psychotherapy in clinical lycanthropy or zoanthropic delusions. Cognitive behavioral therapy is being developed for positive symptoms, however it is mainly studied in persecutory delusion (98, 107). Cognitive behavioral therapy in psychosis may target cognitive biases, for example, jumping to conclusions, and help patients develop alternative explanations to their abnormal experiences or negative affects.

## Limitations

This systematic review is based on case reports, consequently, the level of evidence is low. There is a risk of bias due to the scarcity of case reports in the literature, and the absence of statistical data, as well as the unbalanced number of publications depending on the country. Another limit is that we reviewed the case reports of lycanthropy in any country with no language restriction, but we probably did not access articles in other languages not referenced in Pubmed/Medline or Google Scholar. As there are no neuroscientific studies on zoanthropic syndromes, the reflection remains based on clinical practice, and analogies with other psychiatric syndromes, especially the DMS syndromes. Finally, we limited this review to lycanthropy and kynanthropy, and studying other animal transformation delusions would be complementary.

## Conclusions

Clinical lycanthropy is a rare neuropsychiatric syndrome for which there is little scientific data. It requires complementaristic neuroscientific and cultural approaches.

From a nosological point of view, clinical lycanthropy is a transnosographic syndrome found across diagnostic borders, in schizophrenia, mood disorders, and other psychotic disorders. Thus, clinical lycanthropy is found in the large spectrum of psychotic disorders. Antipsychotic medications may be an efficient psychopharmacological treatment for this rare transnosographic delusional syndrome. In case of depression or mania, the treatment includes antidepressant or mood regulators, respectively.

The neuroscientific hypotheses include the conception of clinical lycanthropy as a cenesthopathy, the conception of clinical lycanthropy as a delusional misidentification of the self-syndrome, impairments of sensory process and integration, impairments of the belief evaluation system, and anomalies in the right hemisphere. This acquired neuroscientific knowledge allows revisiting the mysterious clinical descriptions of early psychiatry. Interestingly, there is a clinical overlap between clinical lycanthropy and other delusional misidentification syndromes, which suggests shared pathophysiology. Today, psychiatrists should exhibit the same precision in describing the neurological anomalies through exams (neuropsychological exams, neuroanatomic, and neurofunctional imagery) as did the earlier psychiatrists in their meticulous clinical observations. Clinical lycanthropy can be considered a culture-bound syndrome. A cultural approach is needed for the clinical assessment of these patients, as well as narrative and patient-centered management. Transtheoretical psychiatric reflections are relevant for some disorders. Neurobiological and cultural perspectives are complementaristic, and future studies should include integrative frameworks.

## Data Availability Statement

The original contributions presented in the study are included in the article/Supplementary Materials, further inquiries can be directed to the corresponding author.

## Author Contributions

SG performed the systematic review and wrote the first draft, with LB and SM. JM improved the draft and supervised the study. MM coordinated the whole study and improved the draft. All authors contributed to the article and approved the submitted version.

## Conflict of Interest

The authors declare that the research was conducted in the absence of any commercial or financial relationships that could be construed as a potential conflict of interest.

## Publisher's Note

All claims expressed in this article are solely those of the authors and do not necessarily represent those of their affiliated organizations, or those of the publisher, the editors and the reviewers. Any product that may be evaluated in this article, or claim that may be made by its manufacturer, is not guaranteed or endorsed by the publisher.
