# Supplementary material for: Clinical Lycanthropy, Neurobiology, Culture: A Systematic Review
Source: Front Psychiatry. 2021 Oct 11;12:718101. doi: 10.3389/fpsyt.2021.718101 (PMC8542696; doi:10.3389/fpsyt.2021.718101)
Supplement: Supplementary file 1 [file Table_1.docx]

**Table S1. Other zoanthropy case reports**

| **References** | **Animal** | **Diagnosis** | **Country** | **Age** | **Gender** | **Treatment** |
| --- | --- | --- | --- | --- | --- | --- |
| [(Bou Khalil et al. 2012)](https://paperpile.com/c/AjxJU6/nvLvC) | snake | Psychotic depression  (major depressive disorder with psychotic features and recurrent MDD) | Lebanon | 47 | F | Aripiprazole  Escitalopram |
| [(Grover, Shah, and Ghosh 2010)](https://paperpile.com/c/AjxJU6/rduwQ) | pig | Psychotic depression | India | 37 | F | ECT Venlafaxine  Olanzapine |
| [(Petra Garlipp, Apel, and Peschel 2009)](https://paperpile.com/c/AjxJU6/Liud) | wild boar | Alcohol intoxication  Bipolar disorder | Germany | 43 | M | - |
| [(Younis and Moselhy 2009)](https://paperpile.com/c/AjxJU6/iJDFL) | cow | Schizophrenia | Iraq or UAE | - | M | Risperidone |
| [(Gödecke-Koch et al. 2001)](https://paperpile.com/c/AjxJU6/OukOg)  [(P. Garlipp et al. 2001)](https://paperpile.com/c/AjxJU6/c7qi1) | frog | Schizophrenia | Germany | 34 | F | Perazine |
| [(Gödecke-Koch et al. 2001)](https://paperpile.com/c/AjxJU6/OukOg) [(P. Garlipp et al. 2001)](https://paperpile.com/c/AjxJU6/c7qi1) | bee | schizophrenia | Germany | 24 | F | - |
| [(Keck et al. 1988)](https://paperpile.com/c/AjxJU6/SmKR5) | gerbil | psychotic depression, bipolar disorder | USA | 38 | M | Antipsychotic, tricyclic antidepressant |
|  | unspecified | schizophrenia  Organic neurological condition | USA | 31 | M | antipsychotic (treatment refractory) |
|  | unspecified | mania (rapid cycling), bipolar disorder, obsessive-compulsive disorder | USA | 16 | M | antipsychotic |
|  | bird | mania, bipolar disorder | USA | 26 | F | antipsychotic, lithium carbonate |
|  | cat | psychotic depression, bipolar disorder | USA | 37 | F | antipsychotic, tricyclic antidepressant |
|  | cat | major depression, recurrent, atypical psychosis, alcohol abuse  EEG: rare sharp, wave activity right temporal era | USA | 24 | M | antipsychotic, tricyclic antidepressant, carbamazepine  (treatment refractory) |
|  | rabbit | mania, bipolar disorder, factitious disorder with psychological features | USA | 23 | M | antipsychotics, lithium |
|  | tiger | borderline personality disorder, factitious disorder with psychological features | USA | 28 | M | None |
